# Cerebrospinal fluid abnormalities in first- and multi-episode schizophrenia-spectrum disorders: impact of clinical and demographical variables

**DOI:** 10.1038/s41398-021-01751-7

**Published:** 2021-12-08

**Authors:** Tatiana Oviedo-Salcedo, Elias Wagner, Mattia Campana, Anna Gagsteiger, Wolfgang Strube, Peter Eichhorn, Marie-Luise Louiset, Jurjen Luykx, Lot D. de Witte, René S. Kahn, Michael E. Benros, Peter Falkai, Alkomiet Hasan

**Affiliations:** 1 Department of Psychiatry and Psychotherapy, University Hospital, LMU Munich, Munich, Germany; 2 Department of Psychiatry, Psychotherapy and Psychosomatics, Medical Faculty, University of Augsburg, Bezirkskrankenhaus Augsburg, Augsburg, Germany; 3 Institute of Laboratory Medicine, Klinikum der Universität München, Ludwig Maximilians-University Munich, Munich, Germany; 4 Brain Center Rudolf Magnus, Department of Translational Neuroscience, University, Medical Center Utrecht, Utrecht, The Netherlands; 5 Icahn School of Medicine at Mount Sinai, New York, NY USA; 6 Biological and Precision Psychiatry, Copenhagen Research Centre for Mental Health, Mental Health Centre Copenhagen, Copenhagen University Hospital, Copenhagen, Denmark; 7 Department of Immunology and Microbiology, Faculty of Health and Medical Sciences, University of Copenhagen, Copenhagen, Denmark

**Keywords:** Physiology, Diagnostic markers

## Abstract

Multiple lines of evidence indicate that immunological and inflammatory alterations contribute at least in a subgroup to the pathophysiology of schizophrenia. In this retrospective chart review, we investigated whether clinical factors contribute to altered cerebrospinal fluid (CSF) findings in schizophrenia-spectrum disorders. Clinical data from electronic medical records of patients with psychotic disorders (ICD-10: F20-F29) who received routine CSF diagnostics at the Department of Psychiatry and Psychotherapy, LMU Munich, Germany, were included. Chi² tests for dichotomous outcomes and independent *t* tests for continuous outcomes were used to compare differences between groups. A total of 331 patients were included in the analyses (43.2% female and 56.8% male). The mean age was 37.67 years (±15.58). The mean duration of illness was 71.96 months (±102.59). In all, 40% (128/320) were first-episode psychosis (FEP) patients and 60% (192/320) were multi-episode psychosis (MEP) patients. Elevated CSF protein levels were found in 19.8% and elevated CSF/serum albumin ratios (Q_Alb_) in 29.4% of the cases. Pleocytosis was found in 6.1% of patients. MEP patients showed significantly higher mean Q_Alb_ compared with FEP patients (*t*_(304.57)_ = −2.75, *p* = 0.006), which did not remain significant after correcting for age. Q_Alb_ elevation occurred more frequently in men (*X*^2^_(1)_ = 14.76, *p* = <0.001). For treatment resistance, family history, and cMRI alterations, no significant differences in CSF-related outcomes were detected. Our work extends other retrospective cohorts confirming a relevant degree of CSF alterations in schizophrenia-spectrum disorders and shows the difficulty to relate these alterations to clinical and disease course trajectories. More research is needed to develop treatment response predictors from CSF analyses.

## Introduction

Schizophrenia is a severe neuropsychiatric disorder with onset mostly in late adolescence to early adulthood [1] and showing a relapsing disease course in approximately two-thirds of the patients and a chronic-progressive course in many of the cases that often leads to a relevant functional and cognitive impairment [2]. Early recognition of psychosis and a thorough diagnostic to rule out non-idiopathic causes are crucial to assure adequate treatment and the best clinical outcome. Moreover, in order to diagnose a schizophrenia-spectrum disorder both the Tenth Revision of the International Classification of Diseases and Related Health Problems (ICD-10) and the Diagnostic and Statistical Manual of Mental Disorders (DSM-V) require an underlying organic condition to be ruledout as a cause for psychosis.

In the past years, the discovery of diseases such as the *N*-methyl-d-aspartate receptor (NMDAR)-antibody-mediated encephalitis, which can in certain cases present primarily with schizophrenia-like symptoms [3, 4], contributed to the discussion about the recommended diagnostic work-up in first-episode-psychosis (FEP) patients. Whereas the clinical significance of NMDAR antibodies being detected in serum from up to 10% of healthy controls as well as in patients with psychotic symptoms [5] remains elusive, recent evidence suggests higher rates in patients with FEP than in controls [6] which supports the hypothesis that reduced activity of the NMDAR has an important role in schizophrenia. NMDAR antibodies in the CSF, especially when co-occurrent neurological symptoms are present, are highly suggestive of autoimmune encephalitis which is a different disease entity than schizophrenia-spectrum. To which extent both disease entities share common pathomechanisms, is to be investigated.

When diagnosing FEP, first-line assessments include physical and neurological examination, a structured neuropsychological assessment, and laboratory tests, whereas further examinations such as neuroimaging (cMRI or CCT) or cerebrospinal fluid (CSF) analyses are considered second-line assessments reserved for cases with atypical presentation of psychosis or in cases where the first-line assessment raises suspicion of an underlying organic cause [7]. Here, differences between countries regarding the diagnostic work-up of somatic causes of psychotic syndromes resulted in relevant discussions on whether CSF analyses should be offered at least once to every patient with psychosis. For example in the German national schizophrenia guideline, cerebral MRI (cMRI) is recommended in every patient with FEP, whereas CSF analyses are recommended for all cases where clinical, laboratory or instrument-based diagnostic tests indicate a possible secondary cause for psychotic symptoms [8]. The UK-based NICE guidelines provide little information regarding the somatic diagnostic work-up in cases of FEP [9] and the recently published US-based APA guidelines recommend cMRI if indicated based on neurological examination or history [10].

CSF analysis is well established as a standard diagnostic for neurological diseases, especially neurodegenerative, immunological, and inflammatory diseases, where different CSF patterns are suggestive for different disease entities. As reviewed by an international consensus group, a growing body of evidence is available indicating a link between inflammation, immunological dysregulation, autoimmunity, and psychotic disorders similar to the relation to autoimmune psychosis and autoimmune encephalitis, suggesting a similar pathological pathway between psychosis and autoimmune encephalitis [4]. Therefore, especially the different autoimmune central nervous system (CNS)-reactive antibodies-reactive antibodies (e.g., anti NMDA, CASPR2, LGI1) have been frequently proposed as immunological human model systems of psychosis. However, the vast majority of patients with schizophrenia do not fulfill clinical criteria for autoimmune psychosis as defined elsewhere [4], but may also have subtle neuroimmunological dysfunctions of yet unclear clinical significance. Whether these subtle alterations may in the future lead us to an immunological subform of psychotic disorders remains currently elusive. While CSF analyses are the best strategy to investigate inflammatory processes in the CNS, our knowledge remains sparse regarding the prevalence of abnormal CSF findings in people with schizophrenia. The reason is that in contrast to the field of neurology, lumbar punctures and subsequent CSF analyses are still not standard procedures in many psychiatric settings. A recent meta-analysis provided evidence from 112 CSF studies, whereof 32 were case-healthy control studies, showing neuroinflammatory alterations in schizophrenia and affective disorders, such as an increase in total CSF protein, increase in CSF/serum albumin ratio (Q_Alb_), increase in IgG ratio or increase in proinflammatory cytokine levels [11]. Oligoclonal bands (OCB) were reported in up to 12.5% of cases investigating schizophrenia patients, with intrathecal immunoglobulin synthesis in up to 7.2%, but of note, OCBs were overall not investigated in most of the publications [11]. A recent publication analyzed retrospectively CSF findings from 992 patients with affective and non-affective psychoses (456 with schizophreniform syndromes) and showed an increase in WBC count in 4%, OCBs in 10% (in 4% intrathecal OCBs), and an elevated protein concentration in 45%. In total, 8% of the investigated patients showed signs of neuroinflammation (based on findings on increased WBC counts, IgG indices, and/or CSF-specific OCBs) and 18% signs of blood-CSF-barrier (B-CSF-B) dysfunction [12]. B-CSF-B dysfunction might be associated with glutamatergic and inflammatory abnormalities which are presumed to be implicated in the etiology of schizophrenia [13]. This body of evidence suggests that in a subgroup of patients with schizophrenia immunological alterations can be detected in the CSF.

However, little is known whether these alterations have clinical relevance and whether these inflammatory profiles impact disease courses, and longitudinal CSF studies of patients with schizophrenia are lacking [11]. Moreover, evidence regarding the impact of disease trajectories (FEP vs. relapsing course), genetic load, or the presence of treatment resistance on CSF findings is sparse. While longitudinal studies investigating CSF trajectories in schizophrenia with implications on cognitive performance and symptom severity have yet to be conducted, cross-sectional approaches can help to unravel patterns of CSF alterations in different subgroups of patients with schizophrenia. Thus, we performed a retrospective analysis of CSF findings from 331 patients with schizophrenia-spectrum disorders to analyze factors that might be implicated in the emergence of CSF alterations.

## Methods

### Study population and data extraction

We retrospectively screened clinical data from the patient-documentation system of our department and searched for patients with psychotic disorders (ICD-10: F20-F29). Diagnoses were made in the clinical setting by the treating physicians according to the 10th version of the International classification of diseases–manual (ICD-10). We included patients who were treated in our tertiary care hospital (Department of Psychiatry and Psychotherapy, University Hospital, LMU Munich, Munich, Germany) between July 2012 and May 2017 and who received a lumbar puncture with subsequent CSF diagnostics as part of the clinical routine. Patients with organic psychotic disorders (ICD-10: F06.0/F06.1/F06.2) and substance-induced disorders (ICD-10: F1X.5) were excluded from our analyses in order to isolate cases of primary psychosis as defined by clinical judgment. We also screened for major neurological symptoms in the clinical documentation such as epileptic seizures and disturbed consciousness that could indicate a major neurological disease at the time of admission. The decision to perform the lumbar puncture was made by the treating physicians according to best clinical practice. In our psychiatric hospital, CSF analyses are usually offered to every FEP patient and to MEP patients who had not received a CSF analysis in the past as part of the diagnostic work-up. Approval for this retrospective analysis was obtained from the local ethics committee (ref. nr. 463-16 and ref. nr. 18-570). Data were manually extracted from the electronic clinical documentation system of the clinic and retrospectively analyzed for abnormalities in routine CSF parameters. Epidemiological sociodemographic data were collected from the medical records.

### Laboratory parameters and interpretation

Paired CSF and serum samples were analyzed in the Institute of Laboratory Medicine, LMU Munich. For the laboratory results, we collected the raw data of every parameter as well as the binary interpretation of the result is “increased” or “within normal range” according to the laboratory. Q_alb_ were adjusted by age according to the formula: Q_alb_ = (4+age/15) × 10^−3^, age indicated as years [14], and the binary cutoff (elevated vs. not elevated CSF/serum ratio) was based on the age-adjusted reference value in our local laboratory. Detection of oligoclonal IgG bands in CSF and serum was performed on a SEBIA HYDRASYS 2 SCAN FOCUSING semiautomated instrument according to the manufacturer’s instructions, which enables immunofixation and direct detection of OCBs on agarose gels using the HYDRAGEL 9CSF kit (Ref. 4353, Sebia, France). As described in a previous publication [15], CSF-OCB pattern classification was based on two consensus report recommendations [16, 17]. Five types of patterns were proposed with only patterns 2 and 3 representing an intrathecal synthesis of IgG: type 1: normal CSF, type 2: two or more CSF restricted OCB, type 3: CSF restricted OCB and additional, identical OCB in serum and CSF, type 4: identical OCB in CSF and serum, “mirror pattern”, type 5: monoclonal bands in CSF and serum. A “mirror pattern” represents systemic immune reaction with passive transfer of oligoclonal bands from the serum to the CSF through an abnormal blood-brain barrier and does not indicate intrathecal IgG synthesis. Protein and albumin levels were measured via Immunoturbidimetry. White blood cell count (WBC) was assessed manually. We corrected the total WBC in cases where the red blood cell count was equal to or more than 1000 erythrocytes/µl. For every 1000 erythrocytes, one cell was subtracted from the total WBC [18]. We divided the results of the WBC for the analysis in this paper into three thresholds for the definition of pleocytosis: pleocytosis defined as > 4 cells/µl, as > 5 cells/µl, and as > 6 cells/µl. In 124 cases, the CSF was tested for neuronal autoantibodies to rule out autoimmune-mediated encephalitis. In a previous publication, we reported the absence of CSF neuronal autoantibodies in this subsample [19].

### Demographic and clinical data

Demographic variables were extracted and described in detail in Table 1. We extracted the age at the time of the lumbar puncture, and categorical data, such as gender, illegal substance abuse, alcohol, cannabis, and nicotine/tobacco abuse according to ICD-10, diagnosed rheumatic, infectious, or CNS disease, type II diabetes, and thyroid disease. Moreover, we defined whether patients were patients with FEP or with recurrent/multi-episode psychosis (MEP) disease course. To account for treatment resistance as a reference to a less treatment-responsive disease course, we screened data for documented treatment with clozapine (lifetime), since clozapine is the first-line pharmacological strategy for treatment resistance in schizophrenia [20, 21]. Since a positive family history for psychiatric disorders has been described as a prominent biological risk factor for the development of schizophrenia [22], we also assessed the documented family history for psychiatric disorders (including substance use disorders). Finally, we analyzed available cMRI findings (documented by the radiologists and neuroradiologists of the university hospital) to identify any cMRI abnormalities such as white matter lesions, atrophy, or tumor. If several pathologies were reported for a single patient, each pathology was counted separately.

**Table 1:** Demographic and epidemiological description of the cohort.

Variables	*N* total	Mean	SD
Average age at the time of the LP (years ± SD)	331	37.67	15.58
	*N* total	Frequency	
Gender (female/male)	331	143/188	
A family history of psychiatric disorders	315	147/315 (46.7%)	
Illegal substance abuse	324	79/324 (24.4%)	
Alcohol abuse	322	74/322 (23%)	
Cannabis abuse	324	81/324 (25%)	
Tobacco abuse	322	148/322 (46%)	
Diagnosed with rheumatic, infectious, oncologic, or CNS disease	329	63/329 (19.1%)	
Diabetes type II	328	18/328 (5.5%)	
Thyroid disease	329	45/329 (13.7%)	
	*N* total	Mean	SD
Duration of illness at the time of the LP (months ± SD)	277	71.96	102.59
	*N* total	Frequency	
FEP at time of LP	320	128/320 (40%)	
Recurrent psychotic episode	320	192/320 (60%)	
Clozapine lifetime treatment	331	68/331 (20.5%)	
Any cMRI alteration(s)	313	173/313 (55.3%)	
White matter lesion	313	109/313 (34.8%)	
Atrophy	313	24/313 (7.7%)	
Tumor	313	11/313 (3.5%)	
Other	313	91/313 (29.1%)	

*cMRI* cerebal magnetic resonance imaging, *CNS* central nervous system, *FEP* first-episode psychosis, *LP* lumbar puncture, *SD* standard deviation.

### Statistical analyses

Using IBM SPSS for Windows (Version 26) for Windows, descriptive statistics were displayed for dichotomous and continuous data. Chi² tests for dichotomous outcomes and independent *t* tests for continuous outcomes were used to test for group differences (female vs. male gender; FEP vs. MEP; treatment resistance yes vs. no; positive psychiatric family history yes vs. no; any cMRI pathologies yes vs. no). Correction of degrees of freedoms in case of detected inhomogeneities of variance according to Levene’s tests was performed where necessary. To correct for age-related effects, analysis of covariance (ANCOVA) was used where necessary. Fisher´s exact test (two-sided) was used instead of Chi² tests in cases with *n* < 5 in 2 × 2 tables. Cramer’s V was used to test the association strength of two categorical variables in cases of significant effects from the Chi² test. Data are presented as mean ± standard deviation.

## Results

### Demographics

We identified a total of 332 patients with schizophrenia-spectrum disorders who met the inclusion criteria and had a CSF test conducted between July 2012 and May 2017. One patient was excluded from the study after initial inclusion since our main focus was to analyze CSF standard parameters and the patient had >7000 erythrocytes in the CSF sample, which substantially impacts the interpretability of the results [18]. Thus, data from a total of *n* = 331 patients were analyzed. Table 1 shows the demographic and epidemiological description of the cohort. The mean age at the time of the lumbar puncture was 37.67 years (±15.58), with *n* = 143 (43.2%) female and *n* = 188 (56.8%) male patients. The mean duration of illness was 71.96 months (±102.59) at the time of the lumbar puncture. 40% (128/320) of patients were FEP and 60% (192/320) MEP patients at the time of the lumbar puncture. In all, 20.5% (68/331) of patients had a documented history of clozapine use, which we used as a proxy for antipsychotic treatment resistance. Almost half of all patients (148/322, 46%) were active smokers. Around a quarter of all patients showed an active drug abuse of at least one substance other than tobacco (24.4%, 79/324). Similarly, around a quarter of patients (25%, 81/324) showed active cannabis consumption while 23% (74/322) of patients had diagnosed alcohol abuse (ICD-10: F10.x). A minority (5.5%, 18/328) was diagnosed with type II diabetes. In all, 45 out of 329 patients (13.7%) were diagnosed with a thyroid condition. In all, 63 out of 329 patients (19.1%) were diagnosed with a rheumatic, infectious, or oncologic disease or a CNS-affecting condition.

### CSF results in the complete sample

Mean CSF protein level across the sample was 38.82 mg/dl ± 14.99 (*N* = 328). Elevated protein levels were found in 19.8% of the cases. Mean Q_Alb_ was 5.86 ± 2.62 (*N* = 330). Q_Alb_ was elevated in 29.4% of the cases. Mean WBC across the sample was 1.56 cells/µl ± 1.80 (*N* = 329). Pleocytosis with WBC > 4 cells/µl was found in 6.1%, WBC > 5 cells/µl in 3.6% and WBC > 6 cells/µl in 2.4% of all cases (see Table 2).

**Table 2:** CSF findings complete sample.

Variables	*N* total	Mean	SD
Protein level (mg/dl)	328	38.82	14.99
Albumin ratio	330	5.86	2.62
White blood cell count (cells/µl)	329	1.56	1.80
	*N* total	Frequency	
Protein level elevated (yes/no)	328	65/328 (19.8%)	
Albumin ratio elevated (yes/no)	330	97/330 (29.4%)	
Pleocytosis (> 4/µl) (yes/no)	329	20/329 (6.1%)	
Pleocytosis (> 5/µl) (yes/no)	329	12/329 (3.6%)	
Pleocytosis (> 6/µl) (yes/no)	329	8/329 (2.4%)	
OCB (yes/no)	329	122/329 (37.1%)	
OCB intrathecal synthesis (yes/no)	122	39/122 (32%)	
OCB type 2		9/122 (7.4%)	
OCB type 3		29/122 (23.8%)	
OCB not specified		1/122 (0.8%)	

*OCB* oligoclonal bands.

OCBs without further specification were positive in 37.1% of the cases (122/329). To further specify these findings we classified OCB findings according to the Andersson criteria and revealed an intrathecal synthesis (Andersson Type 2 and 3) in 11.9% of all cases (39/329) and 32% of the OCB cases (39/122). In all, 25.2% were mirrored in relation to all cases (83/329) and 68% were mirrored with regard to all OCB cases (83/122). Among the 39 cases that presented an intrathecal IgG synthesis, 9/39 (23.1%) cases were classified as Type 2, of which two were patients with a concurrent diagnosis of multiple sclerosis. In one case, one single oligoclonal band was identified in the CSF, which was interpreted as a positive intrathecal result following standard procedures of the local laboratory due to a very prominent occurrence. The remaining 29/39 (74.4%) of the cases were Type 3 bands (see Table 2), whereas one case only showed one weak band that could not be classified with certainty as Type 3. We decided to include this subject in our analyses since subtle immunological alterations are within the scientific scope of this report.

### First-episode psychosis patients vs. MEP patients

Patients with a multi-episodic psychotic disease course (MEP) showed higher mean protein levels compared to patients with FEP (*t*_(304.1)_ = −2.2, *p* = 0.029). This finding could be owing to age differences between the two groups since protein level is an age-dependent parameter; and when correcting for age using a univariate ANCOVA there was no significant difference between the two groups (F_(1, 314)_ = 2.003, *p* = 0.158). There was no significant difference in the rates of elevated protein levels (*X*^2^_(1)_ = 3.6, *p* = 0.058) between FEP and MEP. Mean Q_Alb_ were significantly higher in MEP compared to FEP patients (*t*_(304.57)_ = −2.75, *p* = 0.006); however, no significant difference could be observed with regard to mean Q_Alb_ after adjusting for age (*F*_(1, 316)_ = 3.043, *p* = 0.082). The frequency of elevated Q_Alb_ showed no significant difference (*X*^2^_(1)_ = 2.405, *p* = 0.121). MEP showed higher mean WBC compared with FEP (*t*_(315.87)_ = −2.02, *p* = 0.044). Comparing the WBC of MEP with FEP patients using the three different thresholds for pleocytosis described above, we did not find any significant differences between MEP and FEP patients in neither of the applied thresholds (all *p* > 0.250). The proportion of general (*X*^2^_(1)_ = 3.57, *p* = 0.059) or intrathecal OCB production (*X*^2^_(1)_ = 0.002, *p* = 0.964) showed no significant difference between MEP and FEP groups (see Table 3).

**Table 3:** First-episode psychosis patients (FEP) vs. multi-episode psychosis patients (MEP).

Variables	*N*	First-episode	Multi-episode	*t*/*Χ*²	*df*	*p*
*Demographics*						
Age at time of LP	128/192	34.43 ± 15.00	40.05 ± 15.67	−3.195	318	0.002^a^
Duration of illness (months)	95/180	11.17 ± 27.06	103.31 ± 112.82	−10.405	215.28	<0.001^a^
Age at onset of disease (years)	112/181	34.34 ± 14.89	31.52 ± 12.91	1.653	210.22	0.100^a^
Gender (f/m)	320	49/79	87/105	1.554	1	0.213^b^
*CSF parameter*						
Protein level (mg/dl)	127/190	36.77 ± 13.12	40.41 ± 16.29	−2.196	304.1	0.029^a^
Protein level elevated (yes/no)	317	19/108	45/145	3.595	1	0.058^b^
Albumin ratio	127/192	5.41 ± 2.28	6.19 ± 2.83	−2.745	304.57	0.006^a^
Albumin ratio elevated (yes/no)	319	32/95	64/128	2.405	1	0.121^b^
White blood cell count (cells/µl)	127/191	1.32 ± 1.37	1.70 ± 2.02	−2.017	315.87	0.044*
Pleocytosis (> 4/µl)	318	6/121	13/178	0.589	1	0.443^b^
Pleocytosis (> 5/µl)	318	3/124	8/183			0.536^c^
Pleocytosis (> 6/µl)	318	1/126	6/185			0.250^c^
OCB (yes/no)	318	38/89	77/114	3.569	1	0.059^b^
OCB intrathecal synthesis (yes/no)	115	12/26	24/53	0.002	1	0.964^b^

^a^Independent *t* test.

^b^*X*² test.

^c^Fisher’s exact test (two-sided) was used in case *n* < 5 in a 2 × 2 table.

*CSF* cerebrospinal fluid, *f* female, *LP* lumbar puncture, *m* male, *OCB* oligoclonal bands.

### Gender

Female and male patients showed significant differences in mean protein levels (*t*_(326)_ = −3.49, *p* = 0.001), with male patients showing higher levels than females. However, there was no significant difference between genders with regard to the frequency of protein level elevations (*X*^2^_(1)_ = 3.57, *p* = 0.059). Male patients showed higher mean Q_Alb_ compared with female patients (*t*_(328)_ = −3.15, *p* = 0.002). Male patients also showed more frequently Q_Alb_ elevations compared to female patients (*X*^2^_(1)_= 14.76, *p* = <0.001). Both protein levels and Q_Alb_ are known as age-dependent parameters that physiologically increase with older age. Confirming the well-known feature that the onset of schizophrenia-spectrum disorders occurs earlier in males, male patients were on average younger than female patients (*t*_(285.76)_ = 3.51, *p* = 0.001). Thus, an age-related effect would not explain the difference detected in the protein analyses. After conducting ANCOVAs for both variables to correct for age, the differences between genders remained significant (protein levels: *F*_(1, 325)_ = 21.28, *p* = < 0.001; Q_Alb_: *F*_(1,327)_ = 21.21, *p* = < 0.001). There were no significant differences between genders in the mean values for WBC (*t*_(327)_ = −0.72, *p* = 0.471). After dividing WBC findings into the three categories for pleocytosis, we could not detect significant differences in the proportion of pleocytosis between groups (all *p* > 0.729). Neither the incidence of OCBs in in the general (*X*^2^_(1)_ = 0.15, *p* = 0.703) nor the intrathecal OCBs production (*X*^2^_(1)_ = 0.014, *p* = 0.905) showed a significant difference between genders (see Table 4).

**Table 4:** Gender.

Variables	*N*	Female (*N* = 143)	Male (*N* = 188)	*t*/*Χ*²	*df*	*p*
*Demographics*						
Age at time of LP	143/188	41.11 ± 16.31	35.06 ± 14.51	3.507	285.76	0.001^a^
Duration of illness (months)	124/153	81.85 ± 118.11	63.93 ± 87.62	1.41	221.54	0.161^a^
Age at onset of disease (years)	128/166	35.15 ± 13.69	30.63 ± 13.47	2.83	292	0.005^a^
*CSF parameter*						
Protein level (mg/dl)	140/188	35.53 ± 13.92	41.27 ± 15.32	−3.49	326	0.001^a^
Protein level elevated (yes/no)	328	21/119	44/144	3.57	1	0.059^b^
Albumin ratio	142/188	5.34 ± 2.36	6.25 ± 2.74	−3.15	328	0.002^a^
Albumin ratio elevated (yes/no)	330	26/116	71/117	14.76	1	<0.001^b^
White blood cell count (cells/µl)	141/188	1.48 ± 1.86	1.62 ± 1.75	−0.721	327	0.471^a^
Pleocytosis (> 4/µl)) (yes/no)	329	8/133	12/176	0.071	1	0.790^b^
Pleocytosis (> 5/µl)) (yes/no)	329	5/136	7/181	0.007	1	0.932^b^
Pleocytosis (> 6/µl)) (yes/no)	329	4/137	4/184			0.729^c^
OCB (yes/no)	329	51/91	71/116	0.146	1	0.703^b^
OCB intrathecal synthesis (yes/no)	122	16/35	23/48	0.014	1	0.905^b^

^a^Independent *t* test.

^b^*X*² test.

^c^Fisher’s exact test (two-sided) was used in case *n* < 5 in a 2 × 2 table.

*CSF* cerebrospinal fluid, *f* female, *LP* lumbar puncture, *m* male, *OCB* oligoclonal bands.

### Documented lifetime treatment with clozapine

To take treatment resistance as a measurement for the severity of disease into account, we screened clinical data for documented lifetime treatment with clozapine and compared these patients with a clozapine-naive cohort. We did not find significant differences in mean protein levels between patients with lifetime clozapine treatment and patients without a documented history of clozapine use (*t*_(326)_ = 1.63, *p* = 0.104). There was no significant difference in the frequency of protein level elevation (*X*^2^_(1)_ = 3.56, *p* = 0.059). Both groups did not significantly differ neither in mean Q_Alb_ (*t*_(328)_ = 1.81, *p* = 0.071), nor in the incidence of Q_Alb_ elevations (*X*^2^_(1)_ = 0.361, *p* = 0.548). Mean WBC showed no significant difference between the two subgroups (*t*_(327)_ = 0.84, *p* = 0.402). There was also no significant difference with regard to pleocytosis rates in any of the three categories described previously (all *p* > 0.102). The rates of OCBs (X^2^_(1)_ = 0.117, *p* = 0.732) and the intrathecal OCBs production (*X*^2^_(1)_ = 1.292, *p* = 0.256) showed no significant difference between clozapine-treated and clozapine-naive patients. Of note, patients with lifetime treatment with clozapine had a significantly longer duration of illness compared to clozapine-naive patients (*t*_(80.98)_ = 3.83, *p* < 0.001) (see Table 5).

**Table 5:** Documented treatment with clozapine lifetime.

Variables	*N*	Clozapine treatment lifetime (*N* = 68)	No documented clozapine treatment lifetime (*N* = 263)	*t*/*Χ*²	*df*	*p*
*Demographics*						
Age at time of LP	68/263	38.75 ± 17.45	37.40 ± 15.08	0.586	94.48	0.559^a^
Duration of illness (months)	66/211	123.53 ± 135.98	55.82 ± 83.74	3.83	80.98	<0.001^a^
Age at onset of disease (years)	65/229	28.94 ± 12.63	33.64 ± 13.87	−2.456	292	0.015^a^
Gender (f/m)	331	29/39	114/149	0.011	1	0.917^b^
*CSF parameter*						
Protein level (mg/dl)	328	41.45_(*N* = 68)_± 17.26	38.13_(*N* = 260)_± 14.30	1.63	326	0.104^a^
Protein level elevated (yes/no)	328	19/49	46/214	3.56	1	0.059^b^
Albumin ratio	330	6.37_(*N* = 68)_ ± 3.05	5.72_(*N* = 262)_ ± 2.48	1.81	328	0.071^a^
Albumin ratio elevated (yes/no)	330	22/46	75/187	0.361	1	0.548^b^
White blood cell count (cells/µl)	329	1.72_(*N* = 68)_ ±1.91	1.52_(*N* = 261)_±1.77	0.839	327	0.402^a^
Pleocytosis (> 4/µl) (yes/no)	329	7/61	13/248	2.667	1	0.102^b^
Pleocytosis (> 5/µl) (yes/no)	329	4/64	8/253			0.279^c^
Pleocytosis (> 6/µl) (yes/no)	329	3/65	5/256			0.369^c^
OCB (yes/no)	329	24/44	98/163	0.117	1	0.732^b^
OCB intrathecal synthesis (yes/no)	122	10/14	29/69	1.292	1	0.256^b^

^a^Independent *t* test.

^b^*X*² test.

^c^Fisher’s exact test (two-sided) was used in case n < 5 in a 2 × 2 table.

*CSF* cerebrospinal fluid, *f* female, *LP* lumbar puncture, *m* male, *OCB* oligoclonal bands.

### Family history for psychiatric disorders

We found no significant difference in mean protein levels between patients with a positive family history for psychiatric disorders compared to individuals without a positive family history for psychiatric disorders (*t*_(310)_ = −0.29, *p* = 0.774). Both groups also did not significantly differ with regard to the frequency of protein level elevation (*X*^2^_(1)_ = 0.11, *p* = 0.736). Neither the mean values for Q_Alb_ (*t*_(312)_ = −0.62, *p* = 0.535) nor the frequency of Q_Alb_ elevation (*X*^2^_(1)_ = 3.25, *p* = 0.072) differed significantly between both groups. Furthermore, we could not detect significant difference with regard to mean WBC (*t*_(311)_ = 0.69, *p* = 0.494). There was also no significant difference in the frequency of pleocytosis in any of the applied thresholds (all *p* > 0.608). There was a weak (*V* = 0.115), but significant difference between the frequency of OCBs without further specification (*X*^2^_(1)_ = 4.13, *p* = 0.042) with patients without a psychiatric family history having more frequently positive OCB findings compared with patients with a psychiatric family history. Regarding intrathecal OCBs, no significant difference was found (*X*^2^_(1)_ = 0.001, *p* = 0.971) (see Supplement Table 1).

### Alterations in cMRI

No significant difference in mean protein levels (*t*_(308)_ = −0.41, *p* = 0.680) between patients with and without cMRI alterations could be observed. Furthermore, no significant difference was detected with regard to mean WBC (*t*_(287.216)_ = 1.78, *p* = 0.077) and Q_Alb_ (*t*_(310)_ = −0.23, *p* = 0.818) between the two groups. As expected, patients with MRI pathologies had a longer duration of psychotic illness (*t*_(228.813)_ = 2.08, *p* = 0.038) and were older at the time of the LP (*t*_(307.915)_ = 5.04, *p* < 0.001). With regard to the frequency of protein level elevation (*X*^2^_(1)_ = 0.025, *p* = 0.875), presence of OCBs without further specification (*X*^2^_(1)_ = 2.76, *p* = 0.097) and Q_Alb_ elevation (*X*^2^_(1)_ = 0.152, *p* = 0.696), no significant difference was detected between the two groups. For pleocytosis, no significant difference was detected in any of the applied thresholds (all *p* > 0.091). Patients with clozapine did not have significantly more frequently cMRI alteration than clozapine-naive patients (*X*^2^_(1)_ = 0.044, *p* = 0.835). For intrathecal OCBs, no significant difference was detected between the two groups (*X*^2^_(1)_ = 2.91, *p* = 0.088) (see Supplement Table 2).

## Discussion

In this retrospective study, we report demographical and clinical data from patients with schizophrenia-spectrum disorders that received CSF diagnostics within the clinical routine in a tertiary care hospital. Our cohort entails a relatively homogenously distributed population with regard to disease progression having 40% of patients diagnosed with FEP and 60% of patients with MEP. Moreover, 68 out of 331 patients (20.5%) were treated at least once with clozapine, suggesting treatment resistance or at least a less treatment-responsive course of the disease. More than a quarter of the patients 97/330 (29.4%) and almost a fifth of the cohort 65/328 (19.8%) showed respectively an elevated Q_Alb_ and elevated protein levels in the CSF, possibly implying a blood-brain-barrier alteration. This frequency is within the ranges reported from the largest available meta-analysis [11] and a bit higher than in the largest available retrospective cohort regarding this outcome variable [12]. It is well-known in the literature that the Q_Alb_ is an age-dependent parameter showing higher levels with increasing age [23]. Differences in Q_Alb_ between FEP and MEP in our cohort might rather be explained by these differences in age than by biological effects.

We cannot rule out that long-term antipsychotic medication might be implicated in B-CSF-B dysfunction. As suggested elsewhere [13], age as a covariate might have a stronger impact on B-CSF-B function compared to antipsychotic treatment which is supported by the finding that no significant difference was observed in the extent of B-CSF-B dysfunction with regard to medication status among schizophrenia patients [12]. Moreover, as our clozapine cohort did not show more B-CSF-B dysfunction compared to all other cases, the hypothesis of antipsychotic-mediated B-CSF-B dysfunction becomes also more unlikely. Interestingly, despite being on average older, male patients were characterized by a significantly higher Q_Alb_ than female patients. This gender difference has been reported in other studies. In a recent study with over 2000 neurological patients and 335 healthy controls, sex-dependent differences were found with male patients showing a higher Q_Alb_ than female patients [24] which could explain the difference in the mean values, but not necessarily the difference in the incidence of abnormal elevated Q_Alb_. In a previous large cohort study of 989 patients suffering from affective and non-affective psychoses, height differences between male and female patients were proposed as a plausible explanation for significantly higher Q_Alb_ values and more frequent Q_Alb_ elevations [25].

We detected OCBs in 122 patients (37.1% of 329) from which 39 (12.1% of 329) pointed towards an intrathecal immunoglobulin synthesis. Of these 39 bands, 9 were type II and 29 were type III bands, while one single band could not be classified to either of the categories. These rates are higher than those presented in the most recent meta-analysis [11] and other large-scale cohorts [12, 26], but in line with one report from 2010 based on 61 cases [27]. Of note, the prevalence of OCBs type 2 and 3 among healthy subjects has been reported to be ~4% [28]. Differences across studies may be explained by different approaches in the laboratories with differing cutoff criteria for the presence of intrathecal oligoclonal bands. Nevertheless, one could conclude that a relevant subgroup of patients with schizophrenia shows signs of an intrathecal immunoglobulin synthesis pointing towards a chronic inflammatory process of the CNS. This may reflect pre- and perinatal immunological processes, comorbid subtle neurological conditions, or an immune-mediated subgroup of schizophrenia. Our WBC analyses were performed with different thresholds for pleocytosis derived from the literature. The range was between 2.4% and 6.1% supporting the idea that an acute immunological dysfunction might be present in a subgroup of schizophrenia patients, but whether this is the cause or consequence of the disease at the timepoint of the LP remains to be unraveled. A pleocytosis of >5 is part of the criteria for autoimmune psychosis [4] and our findings highlight that dependent on the threshold around 5% of schizophrenia patients may have pleocytosis that warrants further investigations. It has to be noted that our analyses might overestimate prevalence rates for CSF alterations due to selection bias since CSF diagnostics was not performed in all patients with ICD-10 diagnosis of schizophrenia-spectrum disorders admitted to our hospital. Given the retrospective design, reasons for not performing lumbar punctures in patients with psychotic symptoms are multiple, including, i.e., unwillingness to receive CSF diagnostics or leaving the hospital against medical advice before further diagnostics were performed. Psychotic disorders have been reported to have an overall pooled incidence of 26.6 per 100,000 person-years (22.0–31.7) [29] and assuming that 5% of these patients may have an acute CNS process results in a magnitude of patients that may benefit from CSF diagnostics.

We hypothesized that patients with a family history of psychiatric illness and those patients with pharmacological treatment resistance may differ in CSF findings pointing towards specific immunological alterations related to genetic or disease trajectories. However, we could not establish any differences in these analyses. Genetics plays a prominent role regarding the immunological hypothesis of schizophrenia [30, 31], but we were not able to show this link with our clinically driven analytic approach. Regarding the disease trajectory of schizophrenia, anti-inflammatory treatment has been suggested as a potential pathophysiology-based treatment of schizophrenia [32]. Thus, we speculated whether patients with lifetime use of clozapine as a proxy for treatment resistance would show more cerebral inflammation than patients without previous or current clozapine treatment. We were not able to establish this relationship, but one must note that we could not provide information regarding treatment response, remission, or recovery status of our patients and thus, our negative findings have to be interpreted with caution. However, our analyses show the difficulty to relate CSF findings to clinical outcomes and highlight the need for longitudinal studies. The same arguments must be defined for our FEP vs MEP comparisons where all CSF differences can be related to the effects of age. Finally, our analyses did not show any biologically meaningful relationship between cMRI pathologies and CSF findings. However, one must keep in mind that cMRI abnormalities in psychoses may not reflect disease-related effects as the same rates of cMRI abnormalities were described for healthy controls and schizophrenia patients in a large-scale cross-sectional analysis [33].

Our approach entails several limitations. We report here a retrospective study and no follow-up data were taken into account, thus causal correlations between parameters could not be established. This uncontrolled design causes relevant problems as discussed elsewhere [12]. Next, we did not present any analyses regarding autoantibodies and novel CSF parameters (e.g., neurotransmitter levels). In 124 cases of this cohort, CSF was tested for antineuronal antibodies as published elsewhere [19] and we did not identify any patients with a positive antineuronal antibody test. Furthermore, as a tertiary care hospital, our sample may have a bias as we have more specialized units that may attract patients with special disease courses that, e.g., do not represent the outpatient setting reality. Next, as previously mentioned, our analyses might overestimate effects due to selection bias. Our work does not include control groups of healthy participants or other psychiatric or neurological disorders and thus we cannot make clear conclusions regarding the specificity of the here presented findings. Of note, given the retrospective design, some of the CSF tests might have been taken by indication, potentially influencing our results. Furthermore, the elevated WBC count >5 cells/µl is the most widely used parameter clinically and indicative of neuroinflammation, whereas many of the other cutoff values might not be sufficiently investigated in healthy control populations to determine the exact level for a pathological cutoff. Finally, our analyses have an exploratory nature and were not corrected for multiple comparisons and sufficient power cannot be assumed for all of the here presented findings. Thus, positive findings must be interpreted with caution and reproduced in independent samples.

To conclude, our work extends other large-scale retrospective cohorts confirming a relevant degree of CSF abnormalities (B-CSF-B dysfunction, pleocytosis, intrathecal immunoglobulin synthesis) in patients with schizophrenia. While our findings point towards immunological processes in a subgroup of schizophrenia patients, the clinical relevance with regard to the outcome and treatment response is still elusive. We were not able to relate the CSF findings to prominent clinical variables such as genetic load, pharmacological treatment resistance, FEP, or cMRI pathologies. Thus, more research is needed to develop disease course and treatment response predictors from CSF analyses. However, our findings highlight the need to consider offering CSF analyses to patients with schizophrenia since a significant proportion of our patients show CSF alterations with yet unclear clinical implications. But furthermore, it highlights the need for more scientific research regarding CSF alterations in psychotic disorders with longitudinal study designs, clinical correlation, and the inclusion of control groups to further elucidate not only the possible contributions of these analyses to better understand the relevance for CSF alterations in the disease onset and worsening of psychosis, but also the potential implications for diagnostic recommendations and novel treatment options. Finally, one should note that far more parameters beyond routine CSF analyses can be performed such as neopterin and cytokine measurements, assessments of neurotransmitters, autoantibodies, or microRNA. Such analyses may in the future help to understand the clinical relevance of the here observed CSF alterations.

## Supplementary information

Suppl. Table 1

Suppl. Table 2
