# Supplementary material for: Cerebrospinal fluid abnormalities in first- and multi-episode schizophrenia-spectrum disorders: impact of clinical and demographical variables
Source: Transl Psychiatry. 2021 Dec 8;11:621. doi: 10.1038/s41398-021-01751-7 (PMC8654913; doi:10.1038/s41398-021-01751-7)
Supplement: Supplementary file 1 — Suppl. Table 1 [file 41398_2021_1751_MOESM1_ESM.docx]

| **Variables** | **N** | **Positive family history for psychiatric disorders**  **(N=147)** | **Negative family history for psychiatric disorders**  **(N=168)** | **t/Χ²** | **df** | **p** |
| --- | --- | --- | --- | --- | --- | --- |
| *Demographics* |  |  |  |  |  |  |
| Age at time of LP | 315 | 34.14_(N=147)_ ± 14.61 | 40.17_(N=168)_±15.67 | -3.52 | 313 | <0.001^a^ |
| Duration of illness (months) | 265 | 71.39_(N=119)_±99.72 | 73.73_(N=146)_±107.34 | -0.182 | 263 | 0.855^a^ |
| Age at onset of disease (years) | 281 | 29.20_(N=128)_±12.19 | 34.79_(N=153)_±14.09 | -3.521 | 279 | 0.001^a^ |
| Gender (f/m) | 315 | 61/86 | 78/90 | 0.773 | 1 | 0.379 |
|  |  |  |  |  |  |  |
| *CSF parameter* |  |  |  |  |  |  |
| Protein Level (mg/dl) | 312 | 38.63_(N=145)_± 14.78 | 39.12_(N=167)_± 15.59 | -0.287 | 310 | 0.774^a^ |
| Protein Level elevated (yes/no) | 312 | 30/115 | 32/135 | 0.114 | 1 | 0.736^b^ |
| Albumin ratio | 314 | 5.77_(N=146)_ ± 2.59 | 5.96_(N=168)_ ± 2.70 | -0.622 | 312 | 0.535^a^ |
| Albumin ratio elevated (yes/no) | 314 | 51/95 | 43/125 | 3.246 | 1 | 0.072^b^ |
| White Blood Cell Count (cells/µl) | 313 | 1.61_(N=147)_ ±1.89 | 1.48_(N=166)_±1.54 | 0.685 | 311 | 0.494^a^ |
| Pleocytosis (> 4/µl)) (yes/no) | 313 | 10/137 | 9/157 | 0.261 | 1 | 0.610^b^ |
| Pleocytosis (> 5/µl)) (yes/no) | 313 | 6/141 | 5/161 | 0.263 | 1 | 0.608^b^ |
| Pleocytosis (> 6/µl)) (yes/no) | 313 | 4/143 | 3/163 |  |  | 0.710^c^ |
| OCB (yes/no) | 313 | 45/101 | 70/97 | 4.125 | 1 | 0.042^b^ |
| OCB intrathecal synthesis (yes/no) | 115 | 14/31 | 22/48 | 0.001 | 1 | 0.971^b^ |

**Suppl. Table 1: Positive family history for psychiatric disorders**

^a^ independent t-test, ^b^ X² test, ^c^ Fisher´s exact test (2-sided) was used in case n<5 in a 2x2 table

Abbrev.: CSF=cerebrospinal fluid, f=female, LP=lumbar puncture, m=male, OCB=oligoclonal bands
