# Supplementary material for: Cerebrospinal fluid abnormalities in first- and multi-episode schizophrenia-spectrum disorders: impact of clinical and demographical variables
Source: Transl Psychiatry. 2021 Dec 8;11:621. doi: 10.1038/s41398-021-01751-7 (PMC8654913; doi:10.1038/s41398-021-01751-7)
Supplement: Supplementary file 2 — Suppl. Table 2 [file 41398_2021_1751_MOESM2_ESM.docx]

| **Variables** | **N** | **Patients with cMRI alteration(s)**  **(N = 173)** | **Patients without cMRI alteration(s)**  **(N = 140)** | **t/Χ²** | **df** | **p** |
| --- | --- | --- | --- | --- | --- | --- |
| *Demographics* |  |  |  |  |  |  |
| Age at time of LP |  | 41.13 _(N=173)_ ± 16.81 | 32.84 _(N=140)_ ± 12.27 | 5.037 | 307.915 | <0.001^a^ |
| Duration of illness (months) |  | 77.90 _(N=143)_ ± 113.91 | 54.64 _(N=117)_ ± 62.97 | 2.083 | 228.813 | 0.038^a^ |
| Age at onset of disease (years) |  | 35.59 _(N=154)_ ± 14.77 | 29.11 _(N=124)_ ± 11.58 | 4.101 | 275.826 | <0.001^a^ |
| Gender (f/m) |  | 87/86 | 48/92 | 8.079 | 1 | 0.004^b^ |
|  |  |  |  |  |  |  |
| *CSF parameter* |  |  |  |  |  |  |
| Protein Level (mg/dl) |  | 38.56 _(N=170)_ ± 14.27 | 39.27 _(N=140)_ ± 16.06 | -0.413 | 308 | 0.680^a^ |
| Protein Level elevated (yes/no) |  | 34/136 | 27/113 | 0.025 | 1 | 0.875^b^ |
| Albumin ratio |  | 5.84 _(N=173)_ ± 2.50 | 5.91 _(N=139)_ ± 2.80 | -0.230 | 310 | 0.818^a^ |
| Albumin ratio elevated (yes/no) |  | 59/123 | 43/96 | 0.152 | 1 | 0.696^b^ |
| White Blood Cell Count (cells/µl) |  | 1.71 _(N=171)_ ± 2.12 | 1.36 _(N=140)_ ± 1.29 | 1.777 | 287.216 | 0.077a |
| Pleocytosis (> 4/µl)) (yes/no) |  | 14/157 | 5/135 | 2.859 | 1 | 0.091^b^ |
| Pleocytosis (> 5/µl)) (yes/no) |  | 8/163 | 3/137 |  |  | 0.356^c^ |
| Pleocytosis (> 6/µl)) (yes/no) |  | 6/165 | 1/139 |  | 1 | 0.134^c^ |
| OCB (yes/no) |  | 71/102 | 44/94 | 2.762 | 1 | 0.097^b^ |
| OCB intrathecal synthesis (yes/no) |  | 27/44 | 10/34 | 2.914 | 1 | 0.088^b^ |

**Suppl. Table 2: Any cMRI alteration(s)**

^a^ independent t-test, ^b^ X² test, ^c^ Fisher´s exact test (2-sided) was used in case n<5 in a 2x2 table

Abbrev.: cMRI=cerebral magnetic resonance imaging, CSF=cerebrospinal fluid, f=female, LP=lumbar puncture, m=male, OCB=oligoclonal bands
